# Exercise-induced lactate suppresses ccRCC via CNDP2-mediated depletion of intracellular amino acids

**DOI:** 10.1038/s41420-025-02609-3

**Published:** 2025-07-31

**Authors:** Rui Miao, Chunyan Liu, Yilong Wang, Hui Li, Cuihong Han, Chenchen Wang, Zhizhen Tian, Jiao Liu

**Affiliations:** 1 School of Basic Medicine Sciences, Shandong Second Medical University, Weifang, China; 2 The Department of Pathology, Jining No.1 people’s hospital, Jining, China; 3 School of Medicine, Shandong Xiandai University, Jinan, China; 4 Department of Pathology, Jinshan Hospital, Fudan University, Shanghai, China

**Keywords:** Cancer metabolism, Renal cancer

## Abstract

Although epidemiological evidence has established a link between physical exercise and reduced risk and recurrence of multiple cancers, the association between physical exercise and cancer risk varies depending on the cancer site. Therefore, there is an urgent need to explore the molecular mechanisms underlying the antitumor effects of exercise to identify which types of cancers can benefit the most from physical exercise. Recent studies have shown that amino acids and exercise-induced lactate can be used by Carnosine Dipeptidase 2 (CNDP2) to synthesize lactoyl amino acids (Lac-AAs), which are then immediately secreted. Thus, we propose that lactate can deplete intracellular amino acids in a CNDP2-dependent manner, thereby exerting antitumor effects. Further bioinformatics analysis revealed that clear cell renal cell carcinoma (ccRCC) has the highest CNDP2 expression level among the 33 types of cancer tissues. Therefore, we focused on the inhibitory effects of lactate in this type of cancer and indeed confirmed its inhibitory roles in both cell and animal models. This study not only provides compelling evidence for the anti-cancer effects of physical exercise in ccRCC, but also provides novel insights into the biological role of lactate in the tumor microenvironment and offers potential therapeutic opportunities for mechanism-based treatment of ccRCC.

## Introduction

The global burden of cancer is increasingly becoming a significant concern [1], and studies have shown that nearly half of cancer deaths can be prevented by improving lifestyle habits (such as increasing physical exercise) and reducing environmental risk factors [2]. Scientific evidence indicates that regular physical exercise can effectively reduce the incidence, recurrence, and mortality rates of cancer, and this effect is independent of other risk factors, such as body mass index and smoking status [3, 4].

Epidemiological studies have confirmed that physical exercise can reduce the risk of at least 13 different types of cancer [4]. In addition, a series of animal models have shown that physical exercise can effectively inhibit tumor growth and metastasis [5]. Notably, the protective effects of physical exercise on cancers at different sites vary [6–8]. Therefore, it is crucial to elucidate the molecular mechanisms by which physical exercise affects cancer, which can help us identify which cancer patients are most likely to benefit from physical exercise.

Studies have shown that CNDP2 can catalyze the synthesis of N-lactoyl-amino acids (Lac-AAs) from lactate and amino acids, and that these Lac-AAs are rapidly secreted outside the cell [9, 10]. Based on this finding, we propose that lactate can deplete intracellular amino acids in a CNDP2-dependent manner. Through bioinformatics analysis, we found that ccRCC has the highest expression level of CNDP2, making it the focus of this study. Moreover, ccRCC exhibits active glycolysis, which significantly increases the level of lactate in the tumor microenvironment [11]. This elevated lactate may synergize with the lactate produced by physical exercise to exert an enhanced inhibitory effect on ccRCC. In this study, we attempted to investigate the inhibitory effects of lactate on ccRCC in both cell and animal models.

## Results

### Among the 33 types of cancer tissues, ccRCC exhibits the highest expression levels of CNDP2 and LDHA, and both are positively correlated with patient outcomes

**Fig. 1:**
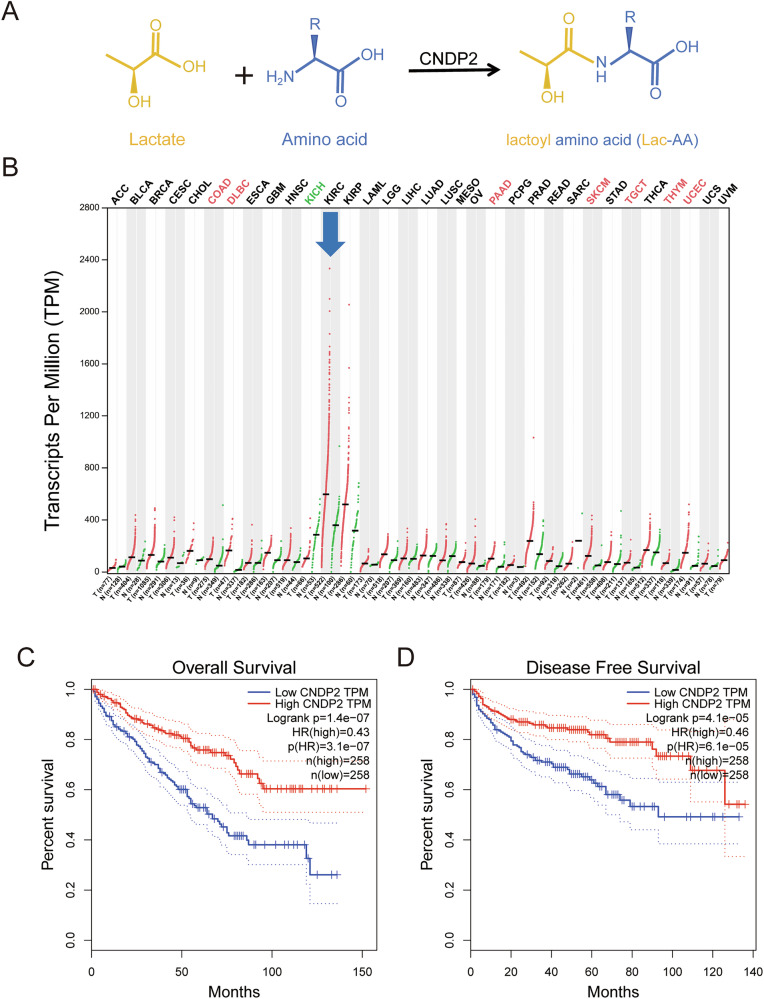
Among 33 types of cancer tissues, CNDP2 has the highest expression level in ccRCC and is positively correlated with patient prognosis. **A** The schematic of the catalytic reaction of CNDP2 in generating lactoyl amino acids. **B** Expression data of CNDP2 in 33 types of cancer and adjacent normal tissues were obtained from the GEPIA database (http://gepia.cancer-pku.cn/index.html). **C**, **D** The correlation analysis of CNDP2 with overall survival (OS) and relapse-free survival (RFS) in ccRCC patients from the GEPIA database is shown (n = 258, ****p < 0.0001).

Recent studies have shown that lactate produced by physical exercise can be catalyzed by CNDP2 to synthesize Lac-AAs with various amino acids [9, 10]. These synthesized Lac-AAs are rapidly secreted and released into the bloodstream (Fig. 1A). Based on this finding, we hypothesize that lactate can deplete the amino acids within CNDP2^+^ cells by inducing the synthesis and secretion of Lac-AAs. The reduction in amino acid levels inhibits cancer cell growth and proliferation through multiple mechanisms, including the well-studied amino acid-sensing complex, the mammalian target of rapamycin complex 1 (mTORC1) [12, 13]. By analyzing the TCGA database in GEPIA (http://gepia.cancer-pku.cn/), we found that among 33 types of human cancers, clear cell renal cell carcinoma (ccRCC) has the highest expression level of CNDP2 (Fig. 1B). Furthermore, the expression level of CNDP2 is positively correlated with the overall survival and disease-free survival of ccRCC patients (Fig. 1C, D). These data suggest that CNDP2 has a significant anti-cancer effect on ccRCC.

As is well established, the Warburg effect is particularly active in ccRCC, resulting in significant lactate accumulation in the tumor microenvironment [11]. To investigate the impact of endogenous lactate on ccRCC, we further analyzed glycolysis and lactate transport-related proteins in the TCGA database of GEPIA and found that the expression level of the key glycolytic enzyme lactate dehydrogenase A (LDHA) in ccRCC is the highest among 33 types of human cancers (S1A). Moreover, the high expression of multiple lactate-related genes is associated with better patient prognosis, suggesting an inhibitory role of lactate in the occurrence and development of ccRCC (S1B) [14].

### Lactate can significantly reduce the intracellular amino acid content in CNDP2^+^ cells

ccRCC accounts for about 75% of renal cell carcinoma (RCC) cases and is the main cause of most RCC-related deaths [15]. Epidemiological studies support that physical exercise can reduce the risk of kidney cancer [6–8], but the molecular mechanisms involved are not yet fully understood. Studies have shown that physical exercise can increase the basal lactate levels in the blood from 0.5–2 mM to 20 mM [16, 17]. Therefore, we propose that lactate produced by physical exercise may be a key factor in its anti-cancer effects in ccRCC, and the accumulation of endogenous lactate due to the “Warburg effect” may further enhance the inhibitory effects of physical exercise on ccRCC.

**Fig. 2:**
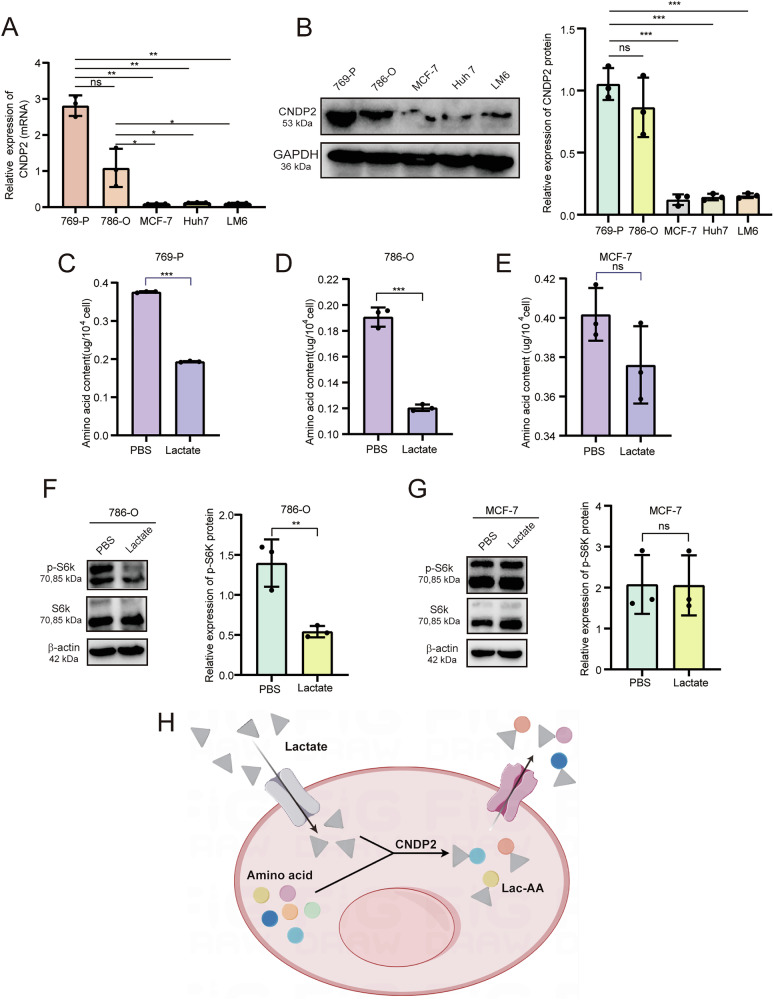
Lactate reduces the intracellular amino acid content of CNDP2^+^ cells. **A**, **B** qRT-PCR and Western blot were used to detect the expression levels of CNDP2 in five types of cancer cells. **C**–**E** The content of amino acids in 769-P, 786-O, and MCF-7 cells was detected after treatment with PBS or Lactate (15 mM) for 24 hours. Data are presented as the mean ± SD and were independently repeated three times. A two-tailed paired Student’s t-test was used for statistical analysis (ns, p > 0.05, *p < 0.05, **p < 0.01, ***p < 0.001). **F**, **G** Western blot was used to detect the phosphorylation level of S6k in two types of cells after treatment with PBS or Lactate for 24 hours. Data are presented as the mean ± SD and were independently repeated three times. A two-tailed paired Student’s t-test was used for statistical analysis (ns, p > 0.05, *p < 0.05). **H** Schematic illustration of lactate-mediated depletion of intracellular amino acids. By Figdraw.

Subsequently, we showed, through RT-qPCR and Western blot, that the mRNA and protein expression levels of CNDP2 in ccRCC cell lines 769-P and 786-O were significantly higher than in other cancer cell lines, including liver cancer cell lines LM6 and Huh 7, and breast cancer cell line MCF-7 (Fig. 2A, B). Therefore, we selected 769-P and 786-O as our subjects of study, with MCF-7 serving as the control cell line. Further experimental results indicated that lactate treatment significantly reduced the intracellular amino acid content in 769-P and 786-O cells, but had no effect on the amino acid content in MCF-7 cells (Fig. 2C–E). Given that mTORC1 is an important intracellular amino acid sensing complex, we further explored the impact of lactate on mTORC1 activity (the level of phosphorylation of its substrate S6K) to confirm the changes in amino acid levels. The results showed that lactate treatment effectively inhibited the activity of mTORC1 in CNDP2^+^ cells 786-O (Fig. 2F), but not in MCF-7 cell (Fig. 2G). For better understanding, we have created a schematic diagram illustrating the mechanism of lactate-mediated intracellular amino acid depletion (Fig. 2H).

### Lactate exerts an inhibitory effect on the proliferation, migration, and invasion of CNDP2^+^ ccRCC cells

**Fig. 3:**
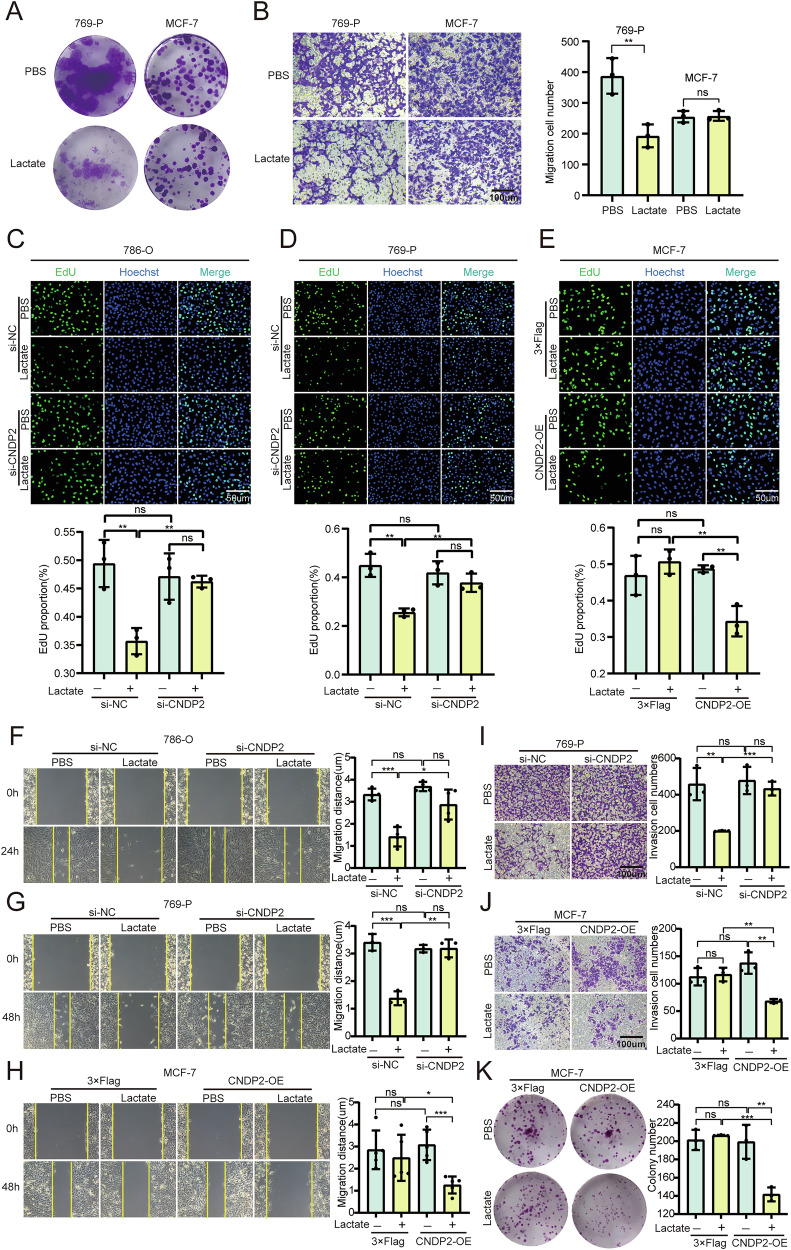
Lactate can inhibit the proliferation, migration and invasion of CNDP2^+^ ccRCC. **A** Results of the colony formation assay in 769-P and MCF-7 cells after the addition of lactate (15 mM), with data independently repeated three times. **B** The Transwell assay was used to evaluate the invasion ability of 769-P and MCF-7 cells (n = 3). Scale bar: 100 µm. **C**–**E** Knockdown or overexpression of CNDP2 in cells, followed by treatment with lactate (15 mM) for 24 hours, cell proliferation ability was assessed using the EdU (green) assay, and nuclear staining was performed with Hoechst (blue). Scale bar: 50 µm. **F**–**H** After knocking down or overexpressing CNDP2, cells were treated with lactate (15 mM), and their migration ability was measured by scratch wound healing assays and their quantitative analysis. Scale bar: 100 µm. **I**, **J** The cell invasion ability was determined by Transwell assay and observed by crystal violet staining, and the cells invading the basolateral side of the chamber were counted. scale bar: 100 µm. **K** After overexpressing CNDP2 in MCF-7 cells and treating with lactate, cell proliferation ability was determined using the colony formation assay. Data were analysed using two-tailed unpaired Student’s test (ns, P > 0.05, *P < 0.05, **P < 0.01, ***P < 0.001, ****P < 0.0001).

We employed various experimental methods to repeatedly confirm the inhibitory effect of lactate on ccRCC cell lines, and the results indicated that the inhibitory effect of lactate on ccRCC cells is CNDP2-dependent. We used the CCK8 assay to determine the half-maximal inhibitory concentration (IC50) values for 786-O, 769-P, and MCF-7 cells, which were found to be 16 mM, 17 mM, and 24 mM, respectively. These findings suggest that lactate exerts a stronger inhibitory effect on CNDP2-positive cells and also provides a reference for the dosing concentration in subsequent experiments. Clone formation and transwell invasion assays further demonstrated that lactate has a stronger inhibitory effect on the proliferation and invasion of CNDP2^+^ cells (Fig. 3A, B, and S2A). Subsequently, we designed siRNA and plasmid of CNDP2 (S2B, C). After verification, we found the knockdown effect of si-CNDP2-2 was the most significant, so we used it for knockdown in subsequent experiments. The results showed that lactate could inhibit the proliferation, migration, and invasion of 786-O and 769-P cells, and knockdown of CNDP2 could reverse this effect. Conversely, lactate did not affect the proliferation, migration, and invasion abilities of MCF-7 cells, while overexpression of CNDP2 enabled lactate to exert its inhibitory effect (Fig. 3C–K, and S2D).

### Swimming exercise can effectively curb the progression of ccRCC

**Fig. 4:**
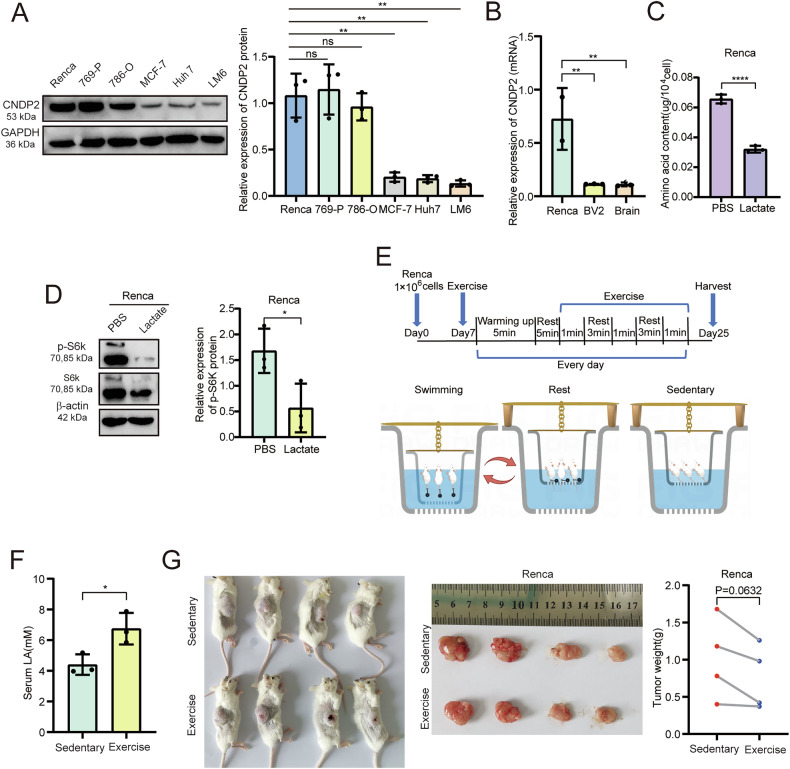
Swimming exercise inhibits the progression of ccRCC. **A** The protein expression levels of CNDP2 in six cancer cells were detected by western blot. **B** The mRNA levels of CNDP2 in mouse renal carcinoma cells Renca, microglia BV2 and brain tissues were detected by qRT-PCR. **C** The contents of amino acids in Renca cells were detected after 24 h treatment with PBS or lactate (15 mM). **D** Western blot was used to detect the phosphorylation levels of S6k in Renca cells after treatment with PBS or lactate for 24 hours. **E** Schematic of xenotransplantation model constructed by subcutaneous injection of Renca cells in BALB/c mice and the subsequent swimming experiment in mice. By Figdraw. **F** Serum lactate levels in mice under sedentary conditions and after swimming exercise (n = 6). **G** Gross appearance and weight of tumors. Data were analysed using two-tailed unpaired Student’s t test (ns, P > 0.05, *P < 0.05, **P < 0.01, ***P < 0.001, ****P < 0.0001).

Renca is a murine renal cancer cell line that highly expresses CNDP2 (Fig. 4A, B). Lactate treatment significantly reduces the intracellular amino acid content of Renca cells (Fig. 4C) and inhibits the activity of mTORC1 (Fig. 4D). Studies have shown that the level of lactate in the bloodstream significantly increases after physical exercise [18]. To investigate the anticancer properties of lactate produced by physical exercise, we constructed a Renca cell subcutaneous xenograft model in BALB/c mice and conducted a swimming experiment (Fig. 4E) [17]. The results indicated that swimming exercise increased serum lactate levels (Fig. 4F) and effectively inhibits tumor growth in mice (Fig. 4G).

### Lactate administration can effectively inhibit the progression of ccRCC

**Fig. 5:**
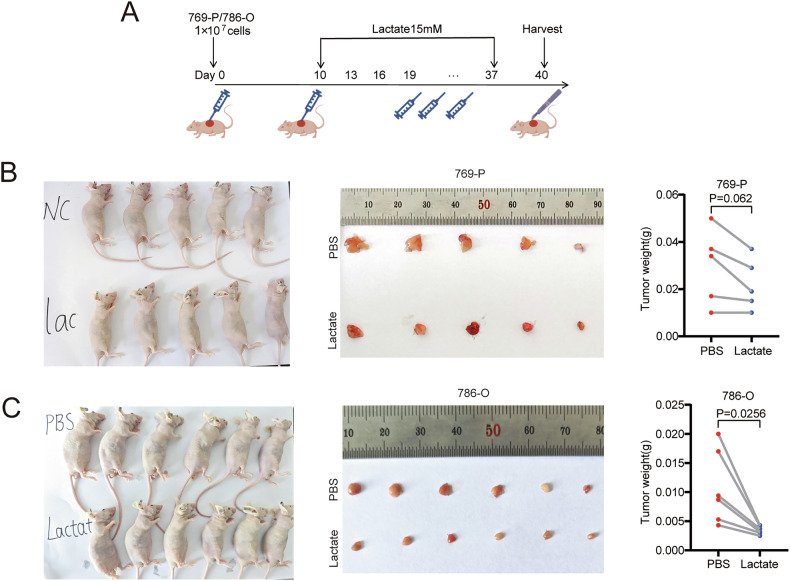
Lactate has an inhibitory effect on ccRCC. **A** Schematic of xenotransplantation model in nude mice and subsequent intratumoral injection of lactate. By Figdraw. **B**, **C** Gross appearance and weight of tumors after injection of 769-P and 786-O cells, respectively. Data were analysed using two-tailed unpaired Student’s t test (ns, P > 0.05, *P < 0.05, **P < 0.01, ***P < 0.001, ****P < 0.0001).

To rule out the impact of other bioactive substances from physical exercise on tumor growth [8], we successfully established a xenograft model by implanting 769-P and 786-O cells subcutaneously into nude mice. Subsequently, we conducted intra-tumoral lactate injection studies to investigate its inhibitory effect on tumor growth (Fig. 5A). The experimental data showed that compared to the control group injected with phosphate-buffered saline (PBS), the tumor weight in the lactate injection group was significantly reduced (Fig. 5B, C).

### Knocking down CNDP2 attenuates the inhibitory effect of lactate on ccRCC

**Fig. 6:**
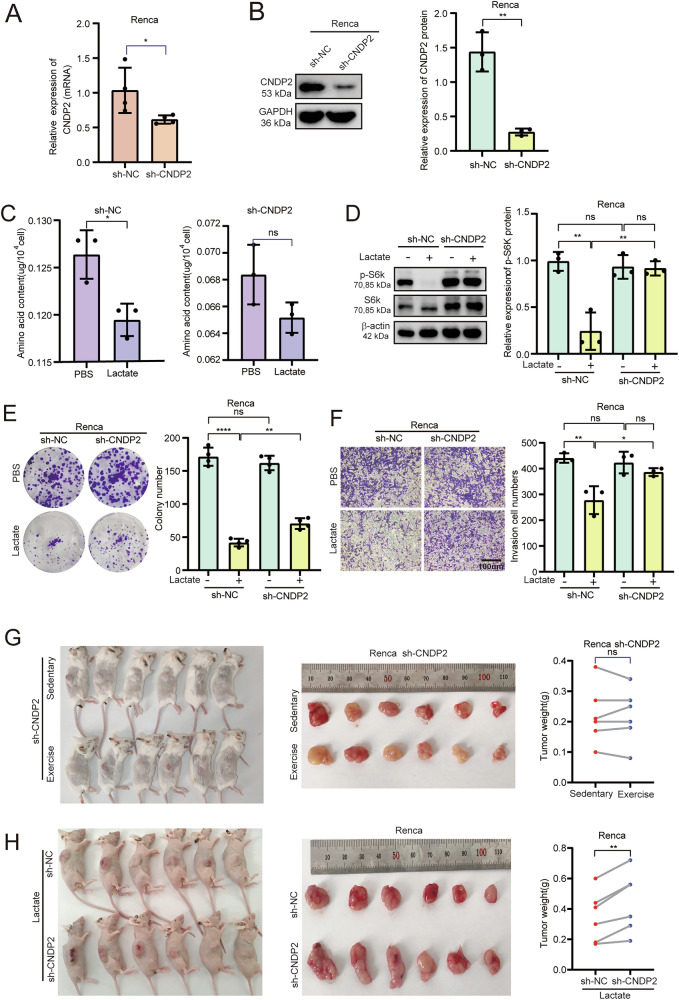
Knockdown of CNDP2 diminishes the inhibitory effect of lactate on ccRCC. **A** qRT-PCR was used to verify the knockdown efficiency of CNDP2 at the mRNA level in the Renca cell line. **B** Western blot was used to verify the knockdown efficiency of CNDP2 at the protein level. **C** After treatment with PBS or lactate (15 mM) for 24 hours, the amino acid content in control cells and CNDP2 stably knocked-down cells was detected. **D** Western blot was used to detect the phosphorylation levels of S6k in sh-CNDP2 cells after treatment with lactate for 24 hours. **E**, **F** Lactate was added to Renca cells with sh-CNDP2 and sh-NC, and cell proliferation and invasion abilities were assessed using the colony formation assay (**E**) and the Transwell assay (**F**). The data were independently repeated three times. Data were analysed using two-tailed unpaired Student’s t test (ns, P > 0.05, *P < 0.05, **P < 0.01, ***P < 0.001, ****P < 0.0001). **G** Using sh-CNDP2 Renca cells, a xenograft model was established by subcutaneous injection in BALB/c mice, which were then divided into a swimming group and a sedentary group. **H** Xenograft models were constructed in BALB/c nude mice using Renca cells with sh-NC and sh-CNDP2, and both groups underwent intratumoral injections of lactate. After tumor harvest, the gross appearance of the mouse tumors was displayed and a statistical analysis of their weights was conducted(n = 6). Data were analysed using two-tailed unpaired Student’s t test (ns, P > 0.05, *P < 0.05, **P < 0.01, ***P < 0.001, ****P < 0.0001).

We next examined the effect of CNDP2 by knockdown with a sh-RNA containing lentivirus in Renca cells (Fig. 6A, B). We found that, compared to the control cells, lactate had no effect on the amino acid content in CNDP2-knockdown cells (Fig. 6C), and it also failed to inhibit the activity of mTORC1 in the cells (Fig. 6D). Additionally, knockdown of CNDP2 had no effect on the cell’s proliferation and invasion capabilities but could diminish the inhibitory effect of lactate (Fig. 6E, F). The results of the mouse swimming experiment indicated that swimming had no inhibitory effect on CNDP2 knockdown ccRCC (Fig. 6G). When lactate was injected into subcutaneous tumors in nude mice, it was found that lactate did not have an inhibitory effect on CNDP2 knockdown ccRCC (Fig. 6H). These results further demonstrate that the inhibitory effect of lactate on ccRCC is CNDP2-dependent.

Based on the series of findings mentioned above, we propose a hypothesis: lactate produced by physical exercise enters CNDP2^+^ ccRCC cells and, together with amino acids, is catalyzed to synthesize Lac-AAs molecules, which are then rapidly released from the cells, leading to the depletion of amino acids within ccRCC cells. The effects of amino acid deprivation on the growth, proliferation, and migration of ccRCC are multifaceted, including but not limited to the mTORC1 signaling pathway [19].

## Discussion

Physical exercise has a broad range of anti-cancer effects, but its efficacy varies significantly depending on the site of cancer onset, and can even be opposite. As an important metabolite of physical exercise, lactate was once considered a metabolic waste product, but later it was found to act as a signaling and energy substance, playing a role in promoting cancer in various types of cancer [20–22]. The lactate shuttle theory posits that lactate can shuttle between “producers” and “consumers”. For example, during physical exercise, lactate produced by skeletal muscle cells (which act as “producers”) can elevate serum lactate concentrations up to 20 mM. This lactate can then enter various organs such as the brain, heart, liver, and kidneys, where it is utilized by these “consumers” [16, 23]. In this manuscript, we propose a novel view of the anti-cancer role of lactate in ccRCC and present experimental data and molecular mechanisms to support this perspective. We speculate that lactate can deplete amino acids in CNDP2^+^ ccRCC cells, thereby inhibiting the mTORC1 signaling pathway and exerting an anti-cancer effect. In this study, we found that swimming exercise effectively suppressed the growth of ccRCC. Considering that physical exercise is a highly complex physiological stimulus, we further performed intratumoral injections in tumor-bearing nude mice to exclude potential confounding effects from other factors on lactate-mediated ccRCC inhibition. The results similarly demonstrated a significant suppressive effect, further confirming that the anti-tumor effect of exercise on ccRCC is at least partially mediated through lactate-dependent molecular mechanisms. Our work provides a theoretical basis for ccRCC patients to choose appropriate exercise methods, lactate injection, and their combined application with drug therapy. For example, the safety and feasibility of lactate injection, as well as its combination with other small-molecule drugs, are particularly relevant for patients with exercise limitations and warrant further exploration in the future. These findings not only expand our understanding of the role of lactate in tumor biology but also provide important scientific evidence for the development of new anti-cancer strategies and the improvement of the quality of life for ccRCC patients.

It is noteworthy that the reduction of intracellular amino acids can trigger a variety of cellular responses. In addition to the mTORC1 signaling pathway mentioned previously, multiple other responses are also involved. For example, decreased translation efficiency can inhibit cell growth [19]. Methionine deficiency can reduce the methylation of cGAS, prompting its translocation from the nucleus, thereby activating the cGAS-STING pathway and exerting anti-tumor immune effects [24]. Furthermore, the insufficiency of amino acids can lead to the interruption of ribosome-associated quality control (RQC) pathways, causing ribosomes to collide and aggregate within the cell, which in turn prompts cGAS to move from the nucleus to the cytoplasm and bind to these collided ribosomes. This promotes cGAS-dependent Interferon-stimulated genes (ISGs) expression and the sustained activation of the cGAS-STING pathway [25]. This cascade ultimately activates innate immune signaling, thereby revealing the key role of amino acid depletion in modulating immune responses. Studies have also indicated that under conditions of amino acid deficiency, proteasomes migrate from the nucleus to the cytoplasm, thereby promoting the degradation of proteins. Additionally, tyrosine, tryptophan, and phenylalanine, which have recently been identified as mTOR activators, function through Sestrin3, and their absence can inhibit cell migration and even induce cell death [26]. In addition, other studies have shown that under amino acid deprivation conditions, p27 promotes autophagy by regulating the autophagy-lysosome pathway, thereby coordinating the cell cycle and growth [27].

A substantial body of research indicates that lactate plays a role in promoting cancer through various mechanisms in multiple tumors [22, 28]. Particularly, recent studies have shown that lactylation occurring on key proteins such as p53 and NBS1 is involved in the occurrence, development, and drug resistance of tumors [21, 22]. These results collectively suggest that inhibiting the production of lactate, particularly by targeting lactate dehydrogenase A (LDHA), is a potential cancer treatment strategy. However, the results of our study strongly suggest that lactate exhibits a significant inhibitory effect in ccRCC. It is worth noting that ccRCC is characterized by a highly glycolytic tumor microenvironment. Moreover, elevated expression of multiple key glycolytic pathway proteins has been associated with improved patient prognosis, further supporting lactate’s inhibitory role in ccRCC progression. These results are consistent with our in vitro experimental findings. Therefore, we propose that inhibiting lactyltransferases, such as AARS1 [29], rather than lactate synthesis itself, may lead to a more potent inhibitory effect in the treatment of ccRCC.

The concept of ‘starving cancer to death’ has prompted major research initiatives [19]. Since glucose has a variety of important physiological roles, therapeutic strategies targeting glucose metabolism often result in broad toxicity, making it difficult to translate into clinical treatment [30]. Unlike glucose, restricting amino acid metabolism may selectively target highly proliferative cancer cells [19]. Studies have shown that at least 14 amino acids are involved in the synthesis of Lac-AAs, six of which are essential amino acids [9, 10, 31]. That is to say, these amino acids can all be pumped out of the cell in a CNDP2-dependent manner by lactate, leading to a deficiency of amino acids. Indeed, our study demonstrates that lactate can deplete intracellular amino acids and strongly inhibit the proliferation of ccRCC cells. Our study provides novel insights into targeting amino acid metabolism to starve ccRCC cells.

In conclusion, this paper proposes and proves that lactate produced by physical exercise enters the cell, where it can be synthesized with amino acids into Lac-AAs under the catalysis of CNDP2 and then be expelled from the cell. This process leads to a decrease in intracellular amino acid content and inhibition of cancer cell growth. This not only expands the understanding of the molecular mechanisms by which physical exercise inhibits cancer, but also clarifies the new biological role of lactate in the tumor microenvironment, and provides new clues for the treatment of ccRCC.

## Methods and materials

### Bioinformatic analysis

Based on the GEPIA (Gene Expression Profiling and Interactive Analysis, http://gepia.cancer-pku.cn/) platform of the TCGA database, we analyzed the expression of CNDP2 in 33 types of cancer tissues and their adjacent normal tissues. In the “Survival Plots” module, we entered the gene name CNDP2, selected KIRC (Kidney Renal Clear Cell Carcinoma) as the cancer type, and then chose OS (Overall Survival) and DFS (Disease-Free Survival) to further analyze the relationship between CNDP2 expression and the survival of KIRC patients. We also analyzed the expression of LDHA in 33 types of cancer tissues and adjacent normal tissues, and investigated its expression in relation to the survival of KIRC patients. In addition, we also analyzed the correlation between the expression levels of glycolysis and lactate transport-related proteins and the overall survival rate of ccRCC patients in the GEPIA database.

### Cell lines and culture

ccRCC cell lines 786-O(CRL-1932), 769-P(CRL-1933) and Renca (CVCL2174) were purchased from Shanghai Fuheng Biotechnology Co., Ltd., and cultured in RPMI-1640 (Gibco C11875500BT). MCF-7 (CL-0149)and Huh 7 (CL-0120)were purchased from Wuhan Pricella Biotechnology Co., Ltd., and cultured in high glucose DMEM (Gibco C11995500BT). Cells were maintained in the cell culture medium supplemented with 10% fetal bovine serum(FBS), 100 U/ml penicillin, and 100 mg/ml streptomycin in a humidified incubator at 37 °C and 5% CO_2_. All cell lines were subjected to short tandem repeat (STR) profiling analysis.

### Amino acid (AA) content assay (Micromethod)

Transplanted cells were placed into a T75 culture flask at a density of approximately 2.5 × 10^6^ cells/ml and incubated overnight in an incubator set at 5% CO_2_ and 37 °C. After the cells adhered, 15 μM lactate was added and the cells were treated for 12 hours. The cells were collected into a centrifuge tube, centrifuged at 1000r/5 min, and the supernatant was discarded. The cell density was adjusted to 5 × 10^6^ cells/ml. Reagent I, 1 ml, was added to resuspend the cells, which were then subjected to ultrasonic cell breaking (in an ice bath, at 20% power, with 3 seconds of sonication and a 10-second interval, repeated 30 times). The cells were centrifuged at 8000 × *g* for 10 min at 4 °C, and 200 μL of the supernatant was transferred to a 1.5 ml EP tube. Reagent II, 200 μL, was added and the tube was tightly capped and placed in a boiling water bath for 15 min, then cooled to room temperature for measurement. A plate reader was preheated for 30 minutes with the wavelength set to 570 nm. In a 1.5 ml EP tube, 20 μL of the liquid to be measured, 200 μL of Reagent II, 200 μL of Reagent III, and 20 μL of Reagent IV were added, mixed well, covered tightly, and kept warm in a boiling water bath for 15 min. After cooling, the EP tube was inverted repeatedly, and the absorbance value at 570 nm was measured, recorded as A test tube. For three sets of sub-wells, 100 μL of A test solution was added to each and then measured.

$$[{\rm{Amino}}\,{\rm{acid}}\,{\rm{content}}\,\left(\frac{\upmu{\rm{g}}}{1{0}^{4}cell}\right)=740.8\times ({\rm{A}}\,{\rm{determination}}-0.0737)\div\,{\rm{cell}}\,{\rm{number}}]$$

The amino acid quantification assay employed in this study is based on the ninhydrin colorimetric principle: The α-amino groups of amino acids react with ninhydrin to form a characteristic blue-violet complex, which exhibits maximal absorbance at 570 nm. The amino acid content can be quantified by measuring the absorbance at this specific wavelength. This method is capable of detecting all proteinogenic amino acids except proline and hydroxyproline.

### qRT-PCR

Total RNA was extracted from cells using the Total RNA Extraction Kit (Beijing Solarbio Science & Technology Co., Ltd.), and the total RNA was then reverse transcribed into cDNA using the Reverse Transcription Kit (FSQ-101, TOYOBO Life Science). mRNA levels were quantified using quantitative real-time PCR (qRT-PCR), with the housekeeping gene β-actin selected as the reference gene. The machine’s reaction conditions were set as follows: Step 1, 95 °C for 60 seconds. Step 2, 95 °C for 15 seconds, 60 °C for 15 seconds, and 72 °C for 45 seconds, repeated for 39 cycles. Step 3, a melting curve analysis was performed. The reaction system was prepared according to the instructions provided with the TOYOBO company’s mix and mixed thoroughly. The mixture was centrifuged for 3 minutes. After the reaction was complete, the data was organized. The relative expression of genes was analyzed using the appropriate formula. The primer sequences are shown in Table 1.

**Table 1:** Primers designed for RT-PCR.

Gene		Sequence
Human CNDP2	Forward primer	GACGACATCGACTTTGACATAG
	Reverse primer	CAGAGAAGGCGCCTTCGATG
Mouse Cndp2	Forward primer	CAGACATGATACCGGAAGTTG
	Reverse primer	CCTTCTCTGGTCAAGTCAGG
Human β-actin	Forward primer	GTGGGGCGCCCCAGGCACCA
	Reverse primer	CTCCTTAATGTCACGCACGATTTC
Mouse β-actin	Forward primer	TTTCCAGCCTTCCTTCTTGGGTATG
	Reverse primer	ATAGAGGTCTTTACGGATGTCAACG

### Protein extraction and Western blot analysis

Protein extraction was performed using RIPA Tissue/Cell Lysis Solution (Cat#R0020; Beijing Solarbio Science & Technology Co., Ltd.) and PMSF (Cat#P0100). The RIPA solution and PMSF were added to the cells in a 100:1 ratio (RIPA to PMSF) and thoroughly mixed. The cells were then scraped using a cell scraper tool and completely lysed.

Equal amounts of protein (10 μl) were loaded onto a 10% polyacrylamide gel, separated by SDS-PAGE, and transferred onto a PVDF membrane. The PVDF membrane was blocked with a solution of 5% skim milk and 0.05% Tween in 1× TBST for 2 hours, then incubated with the primary antibody overnight at 4 °C. Subsequently, the corresponding secondary antibodies were prepared at a ratio of 1:5000 and incubated for 1.5 hours at room temperature. Finally, an Enhanced Chemiluminescence (ECL) Reagent (Cat#ELC-F-100) was used, with reagent A and B mixed in a 1:1 ratio. The PVDF membrane was placed in an exposure apparatus for band imaging, with the luminescent solution added to the top of the bands, and the bands were analyzed using ImageJ software. The following antibodies were used: CNDP2 (1/1000, CST, no. 14925-1-AP, Proteintech); β-Actin (1/5000, CST, no. 20536-1-AP, Proteintech); GAPDH (1/20000, CST, no. 10494-1-AP, Proteintech); S6k (1/1000, CST, no. 2708, Cell Signaling); phospho-S6k (1/1000, CST, no. 9234, Cell Signaling).

### Cell immunofluorescence assay

Glass slides with adherent cells in a culture dish were washed with PBS, and then the slides were fixed with a 4% paraformaldehyde (PFA) solution. The slides were permeabilized with 0.5% Triton X-100 at room temperature for 20 minutes. Normal goat serum was applied to the slides, which were incubated at room temperature for 30 minutes to block non-specific binding. The primary antibody (Ki67) was added to the slides, which were then placed in a humidified chamber and incubated overnight at 4 °C. The fluorescent secondary antibody was added, and it is important to note that all subsequent steps should be performed in the dark or under minimal light conditions to prevent bleaching of the fluorescent signal. For nuclear counterstaining, DAPI was applied and the slides were incubated in the dark for 5 minutes to stain the nuclei. The slides were gently blotted with absorbent paper to remove excess liquid, and then mounted using an anti-fade mounting medium that contains a fluorescence quencher. Finally, the slides were examined and images were captured under a fluorescence microscope.

### Plasmid construction and transfection

We amplified the full-length cDNA encoding the CNDP2 gene by PCR. Subsequently, the purified CNDP2 product was ligated into the P3xFlag-CMV-7.1 vector according to the manufacturer’s instructions. The si-RNA was synthesized by Genomeditech (Shanghai) Co., Ltd. Transfection was performed using Lipofectamine reagent (Thermo Fisher Scientific, USA) according to the manufacturer’s instructions. After incubating the transfection mixture for 6–8 hours, the cells were cultured with fresh medium containing 10% FBS for subsequent experiments. The mouse CNDP2 gene interference lentivirus (sh-CNDP2) and the negative control lentivirus (sh-NC) were purchased from VizGene Biotechnology Co., Ltd. Renca cells were seeded into 6-well plates at a density of 3 × 10^5^ cells per well and incubated overnight in a CO_2_ incubator at 37 °C and 5% CO_2_. After the cells adhered, the original medium was discarded. Based on the optimal MOI (multiplicity of infection) value determined from preliminary experiments, the required amount of virus was calculated, and the virus (7 × 10^8^ TU/ml, 60 μL) was added to fresh medium for transduction. Following lentiviral transduction, transduced cells were selected with puromycin. The cells were cultured in medium (RPMI-1640) supplemented with 2 μg/ml puromycin (Thermo Fisher Scientific, USA) for 5 days. Successfully transduced cells were identified by observing GFP expression under a fluorescence microscope. The sequences of si-RNA and sh-RNA used in the transfection are detailed in Table 2.

**Table 2:** Sequences of si-RNA and sh-RNA.

Gene	Sequence (5’-3’)
Human CNDP2-si-1	CUACCUAACUAAGAAGUUUtt AAACUUCUUAGUUAGGUAGtt
Human CNDP2-si-2	CGACUUUGACAUAGAGGAGUUtt AACUCCUCUAUGUCAAAGUCGtt
Human CNDP2-si-3	GCAGCAACAAAGACCUCCAUUtt AAUGGAGGUCUUUGUUGCUGCtt
CNDP2-sh-1	CGACCACATTGACTTCGATATTTCAAGA GAATATCGAAGTCAATGTGGTCGTTTTTT
CNDP2-sh-2	GACCGCTACGTCAAGAAACTTCTCGAG AAGTTTCTTGACGTAGCGGTCTTTTTT
CNDP2-sh-3	GGCCATGACGGATCTCATTTCTTCAAG AGAGAAATGAGATCCGTCATGGCCTTT TTT
sh-NC	TTCTCCGAACGTGTCACGTTTCAAGAG AACGTGACACGTTCGGAGAATTTTTT

### EdU assay

Cell proliferation was assessed using the EdU Cell Proliferation Kit (C0071S, Beyotime, Shanghai, China). Transfected cells (5000 cells/well) were cultured with lactate in a 96-well plate for 24 hours, then the culture medium was removed and 10 μL of EdU working solution (final concentration of 10 μM) was added to each well, followed by incubation at 37 °C for 2 hours. Subsequently, the cells were fixed with 4% paraformaldehyde at room temperature for 15 minutes, and then incubated with the prepared Click reaction solution in the dark for 30 minutes. The cells were then washed three times with QuickBlock™ Immunostaining Blocking Buffer P0260 (Beyotime) for 5 minutes each. After incubation with Hoechst 33342 at room temperature in the dark for 10 minutes, the cells were rinsed three times with the washing solution. The total number of cells and the number of EdU-positive cells in five randomly selected fields of view under an optical inverted microscope (Carl Zeiss AG, Germany) were used to calculate the percentage of EdU-positive cells (%).

### Cell counting kit (CCK)-8 assay

Cell viability assessment was performed using CCK-8 kits (C008-2, Shanghai Qihai Futai Technology Company). A 96-well plate was prepared, and 100 μL of culture medium containing 1 × 10^3^ cells was added to each well. Five replicate wells were used for each condition. The plate was then placed in a cell incubator. After overnight incubation, lactate was added for 24, 48, 72 and 96 hours, the culture medium containing lactate was removed, and the CCK-8 working solution was prepared with the culture medium at a ratio of 10:1. 100 μl of the working solution was added to each well, incubated for 2 hours, and then the absorbance value at 450 nm was measured with a microplate reader (Thermo Company, USA) for data analysis.

### Transwell assay

The 6.5 mm diameter, 8.0 μm pore size Transwell chambers (BIOFIL, Guangzhou, China) are placed into 24-well plates, and the upper surface of the chamber was covered with 50 μL of Matrigel (C0372, Beyotime, Shanghai, China) to assess invasion, or left untreated to assess migration, followed by an additional 2 hours of incubation in the incubator. Cells that have been treated with lactate for 24 hours (approximately 3 × 10^4^ cells) are added to the upper chamber, and serum-free medium is supplemented up to 200 μL, while 600 μL of complete medium containing 10% FBS is added to the lower chamber through the side ports. After a 24-hour incubation in the incubator, the plate is removed and the non-migrated or non-invaded cells from the upper side of the chamber are gently wiped away with a cotton swab. The cells that have migrated or invaded to the underside of the membrane are fixed in 4% paraformaldehyde for 20 minutes, and then stained with 0.1% crystal violet for 20 minutes. After washing three times with PBS, the inside of the chamber is carefully wiped with a cotton swab to remove any residual cells that might interfere with the experimental results. Cell migration is observed under a microscope at 10× magnification, with three non-overlapping fields of view selected and photographed. The number of migrated or invaded cells is counted using ImageJ software.

### Cell clone formation

The cells were digested into a cell suspension and counted. Four hundred cells were seeded per well in a 12-well plate. The culture medium was replaced with fresh medium every 2–3 days. After approximately 10 days of cultivation, when cell clusters became visible under a microscope, the culture was terminated. The culture medium was removed from the wells, and the cells were gently washed with PBS three times. The cells were fixed with 4% paraformaldehyde at room temperature for 15 minutes, then stained with 0.1% crystal violet for 20 minutes. They were rinsed three times with PBS, air dried at room temperature, and photographed with a microscope. Finally, ImageJ software was used to count the cell colonies.

### Scratch wound-healing assay

Cells were seeded into six-well plates and allowed to adhere. Once they had adhered, transfection was performed. A scratch assay was conducted when the cells reached 80% confluence, 24–48 hours post-transfection. Sterile 200 μL pipette tips were used to make three parallel vertical scratches through the uniform layer of cells, followed by three washes with phosphate-buffered saline to remove cellular debris. Finally, an inverted microscope was used to capture images at 0, 12, 24, and 48 hours to determine cell migration. The wound healing rate was determined by calculating the distance the cells migrated.

$${\Delta }{Scratch}\,{\rm{width}}_{\rm{n}}\,={\rm{SW}}_{\rm{i}}-{\rm{SW}}_{\rm{n}}$$

where SWi denotes the initial temporary storage width and SWn is the scratch width at the n-th hour after the scratch was formed.

### In vivo experiments

#### Xenotransplantation of nude mice

Male BALB/c nude mice (4 weeks old, 18-20 g) were purchased from Jinan Pengyue Laboratory Animal Breeding Co., Ltd. and acclimated to an SPF-level environment for one week. At 5 weeks of age, the nude mice were randomly divided into two groups for subcutaneous tumor implantation (an experimental group injected with lactate and a control group injected with PBS), with six mice in each group. 786-O cells were collected by trypsin digestion and centrifugation, resuspended in PBS, counted, and adjusted to a concentration of 1 × 10^7^/100 μl, with an equal amount of Matrix-Gel^TM^ (C0383-5ml, Beyotime) added. The 1 ml syringe was used to inject the cell suspension into the right dorsal side of each nude mouse, with 200 μl injected per mouse. Model establishment was confirmed (tumor volume ≥100 mm³ by Day 7), the volume of the tumors was measured, and the length (a) and width (b) of the mouse tumors were measured every three days. Lactate and PBS were injected intratumorally into the experimental and control groups, respectively, every three days (lactate diluted to 15 mM with PBS, 100 μl injected per mouse). The tumor volume was calculated using the formula (V) = 1/2ab^2^. At the end of the study, the mice were euthanized. Subsequently, the tumors were harvested, weighed, and prepared for further analysis.

#### Wild mouse swimming test

Male BALB/c mice (6 weeks old, around 20 g) were purchased from Jinan Pengyue Laboratory Animal Breeding Co., Ltd. The hair on the right dorsal side of the mice was shaved using a hair remover. Renca cells were collected by trypsin digestion and centrifugation, resuspended in PBS, counted, and adjusted to a concentration of 1 × 10^6^/100 μl, with an equal amount of Matrix-GelTM added. The cell suspension was injected subcutaneously into the right side of the mice. Seven days after tumor cell injection, swimming experiments were conducted on the experimental group of mice. Mice swam in a barrel-shaped container (38 cm high, 30 cm in diameter) filled with water 20 cm deep (at 23-25 °C). The mice were randomly divided into two groups: swimming group and resting group. The swimming group mice swam freely for 5 minutes (without load) to warm up and then rested for 5 minutes. Subsequently, the mice underwent tail-loaded swimming, with a load of 15% of their body weight, swimming for three sessions of 1 minute each (with a 3-minute rest in between). Three sets of swimming experiments were conducted each day. Meanwhile, the resting group stood in shallow water, maintaining the same water temperature stimulation conditions for each group of mice. Mice were excluded if they met humane endpoints (>20% body weight loss, tumor ulceration). Twenty days after tumor implantation, the mice were euthanized and the tumors were harvested.

#### Serum lactate (LA) assay

Serum LA levels were determined using a commercial assay kit according to the manufacturer’s instructions (A019-2-1, Nanjing Jiancheng Bioengineering Institute, China).

### Statistical analysis

All data were included in the statistical analysis conducted using GraphPad Prism 8.0 software. An unpaired Student’s t-test (two-tailed) was used for comparisons between two unpaired groups. Data obtained from repeated experiments are presented as the mean ± standard deviation (SD), with the levels of statistical significance set at *p < 0.05, **p < 0.01, ***p < 0.001, ****p < 0.0001; p < 0.05 was considered statistically significant.

## Supplementary information

Full and uncropped western blots

Supplementary Figure Legend

## Data Availability

The datasets used in the analysis can be obtained by contacting the corresponding author on reasonable request.

## References

[1] Sung H, Ferlay J, Siegel RL, Laversanne M, Soerjomataram I, Jemal A, et al. Global cancer statistics 2020: globocan estimates of incidence and mortality worldwide for 36 cancers in 185 countries. CA Cancer J Clin. 2021;71:209–49. 33538338 10.3322/caac.21660

[2] Tran KB, et al. The global burden of cancer attributable to risk factors, 2010-19: a systematic analysis for the global burden of disease study 2019. Lancet 2022;400:563–91. 10.1016/S0140-6736(22)01438-6 PMC9395583 35988567

[3] Stamatakis E, Ahmadi MN, Friedenreich CM, Blodgett JM, Koster A, Holtermann A, et al. Vigorous intermittent lifestyle physical activity and cancer incidence among nonexercising adults: the UK biobank accelerometry study. JAMA Oncol. 2023;9:1255–9. 37498576 10.1001/jamaoncol.2023.1830 PMC10375384

[4] Moore SC, Lee I, Weiderpass E, Campbell PT, Sampson JN, Kitahara CM, et al. Association of leisure-time physical activity with risk of 26 types of cancer in 1.44 million adults. JAMA Intern Med. 2016;176:816–25. 27183032 10.1001/jamainternmed.2016.1548 PMC5812009

[5] Ashcraft KA, Peace RM, Betof AS, Dewhirst MW, Jones LW. Efficacy and mechanisms of aerobic exercise on cancer initiation, progression, and metastasis: a critical systematic review of in vivo preclinical data. Cancer Res. 2016;76:4032–50. 27381680 10.1158/0008-5472.CAN-16-0887 PMC5378389

[6] Lavery JA, Boutros PC, Knight D, Tammela T, Moskowitz CS, Jones LW. Association of exercise with pan-cancer incidence and overall survival. Cancer Cell. 2024;42:169–71. 38181796 10.1016/j.ccell.2023.12.007 PMC11369973

[7] Hojman P, Gehl J, Christensen JF, Pedersen BK. Molecular mechanisms linking exercise to cancer prevention and treatment. Cell Metab. 2018;27:10–21. 29056514 10.1016/j.cmet.2017.09.015

[8] Fiuza-Luces C, Valenzuela PL, Gálvez BG, Ramírez M, López-Soto A, Simpson RJ, et al. The effect of physical exercise on anticancer immunity. Nat Rev Immunol. 2024;24:282–93. 37794239 10.1038/s41577-023-00943-0

[9] Li VL, He Y, Contrepois K, Liu H, Kim JT, Wiggenhorn AL, et al. An exercise-inducible metabolite that suppresses feeding and obesity. Nature. 2022;606:785–90. 35705806 10.1038/s41586-022-04828-5 PMC9767481

[10] Jansen RS, Addie R, Merkx R, Fish A, Mahakena S, Bleijerveld OB, et al. N-lactoyl-amino acids are ubiquitous metabolites that originate from cndp2-mediated reverse proteolysis of lactate and amino acids. Proc Natl Acad Sci USA. 2015;112:6601–6. 25964343 10.1073/pnas.1424638112 PMC4450436

[11] Courtney KD, Bezwada D, Mashimo T, Pichumani K, Vemireddy V, Funk AM, et al. Isotope tracing of human clear cell renal cell carcinomas demonstrates suppressed glucose oxidation in vivo. Cell Metab. 2018;28:793–800. 30146487 10.1016/j.cmet.2018.07.020 PMC6221993

[12] Ge M, Zhang C, Zhang N, He P, Cai H, Li S, et al. The trna-gcn2-fbxo22-axis-mediated mtor ubiquitination senses amino acid insufficiency. Cell Metab. 2023;35:2216–30. 37979583 10.1016/j.cmet.2023.10.016

[13] Dai X, Yan P, Wei W. Amino acid availability governs mtor ubiquitination. Cell Res. 2024;34:335–6. 38102197 10.1038/s41422-023-00910-3 PMC11061116

[14] Ito K, Suda T. Metabolic requirements for the maintenance of self-renewing stem cells. Nat Rev Mol Cell Biol. 2014;15:243–56. 24651542 10.1038/nrm3772 PMC4095859

[15] Li Y, Lih TM, Dhanasekaran SM, Mannan R, Chen L, Cieslik M, et al. Histopathologic and proteogenomic heterogeneity reveals features of clear cell renal cell carcinoma aggressiveness. Cancer Cell. 2023;41:139–63. 36563681 10.1016/j.ccell.2022.12.001 PMC9839644

[16] Pino RM, Singh J. Appropriate clinical use of lactate measurements. Anesthesiology. 2021;134:637–44. 33332524 10.1097/ALN.0000000000003655

[17] Liu W, Zhang S, Li Q, Wu Y, Jia X, Feng W, et al. Lactate modulates iron metabolism by binding soluble adenylyl cyclase. Cell Metab. 2023;35:1597–612. 37480842 10.1016/j.cmet.2023.06.017

[18] Li X, Yang Y, Zhang B, Lin X, Fu X, An Y, et al. Lactate metabolism in human health and disease. Signal Transduct Target Ther. 2022;7:305. 36050306 10.1038/s41392-022-01151-3 PMC9434547

[19] Pathria G, Ronai ZA. Harnessing the co-vulnerabilities of amino acid-restricted cancers. Cell Metab. 2021;33:9–20. 33406406 10.1016/j.cmet.2020.12.009 PMC7837405

[20] Certo M, Tsai C, Pucino V, Ho P, Mauro C. Lactate modulation of immune responses in inflammatory versus tumour microenvironments. Nat Rev Immunol. 2021;21:151–61. 32839570 10.1038/s41577-020-0406-2

[21] Zong Z, Xie F, Wang S, Wu X, Zhang Z, Yang B, et al. Alanyl-trna synthetase, aars1, is a lactate sensor and lactyltransferase that lactylates p53 and contributes to tumorigenesis. Cell. 2024;187:2375–92. 38653238 10.1016/j.cell.2024.04.002

[22] Chen H, Li Y, Li H, Chen X, Fu H, Mao D, et al. Nbs1 lactylation is required for efficient dna repair and chemotherapy resistance. Nature. 2024;631:663–9. 38961290 10.1038/s41586-024-07620-9 PMC11254748

[23] Brooks GA. The science and translation of lactate shuttle theory. Cell Metab. 2018;27:757–85. 29617642 10.1016/j.cmet.2018.03.008

[24] Fang L, Hao Y, Yu H, Gu X, Peng Q, Zhuo H, et al. Methionine restriction promotes cgas activation and chromatin untethering through demethylation to enhance antitumor immunity. Cancer Cell. 2023;41:1118–33. 37267951 10.1016/j.ccell.2023.05.005

[25] Wan L, Juszkiewicz S, Blears D, Bajpe PK, Han Z, Faull P, et al. Translation stress and collided ribosomes are co-activators of cgas. Mol Cell. 2021;81:2808–22. 34111399 10.1016/j.molcel.2021.05.018 PMC8260207

[26] Livneh I, Cohen-Kaplan V, Fabre B, Abramovitch I, Lulu C, Nataraj NB, et al. Regulation of nucleo-cytosolic 26s proteasome translocation by aromatic amino acids via mtor is essential for cell survival under stress. Mol Cell. 2023;83:3333–46. 37738964 10.1016/j.molcel.2023.08.016

[27] Nowosad A, Jeannot P, Callot C, Creff J, Perchey RT, Joffre C, et al. P27 controls Ragulator and mTOR activity in amino acid-deprived cells to regulate the autophagy-lysosomal pathway and coordinate cell cycle and cell growth. Nat Cell Biol. 2020;22:1076–90. 32807902 10.1038/s41556-020-0554-4

[28] Cheng Q, Shi X, Li Q, Wang L, Wang Z. Current advances on nanomaterials interfering with lactate metabolism for tumor therapy. Adv Sci (Weinh). 2024;11:e2305662. 37941489 10.1002/advs.202305662 PMC10797484

[29] Li H, Liu C, Li R, Zhou L, Ran Y, Yang Q, et al. Aars1 and aars2 sense l-lactate to regulate cgas as global lysine lactyltransferases. Nature. 2024;634:1229–37. 39322678 10.1038/s41586-024-07992-y

[30] Ganapathy-Kanniappan S, Geschwind JH. Tumor glycolysis as a target for cancer therapy: progress and prospects. Mol Cancer. 2013;12:152. 24298908 10.1186/1476-4598-12-152 PMC4223729

[31] Scott B, Day EA, O’Brien KL, Scanlan J, Cromwell G, Scannail AN, et al. Metformin and feeding increase levels of the appetite-suppressing metabolite lac-phe in humans. Nat Metab. 2024;6:651–8. 38499765 10.1038/s42255-024-01018-7 PMC11052712

