# Supplementary figures and images for: Exercise-induced lactate suppresses ccRCC via CNDP2-mediated depletion of intracellular amino acids
Source: Cell Death Discov. 2025 Jul 31;11:356. doi: 10.1038/s41420-025-02609-3 (PMC12310999; doi:10.1038/s41420-025-02609-3)

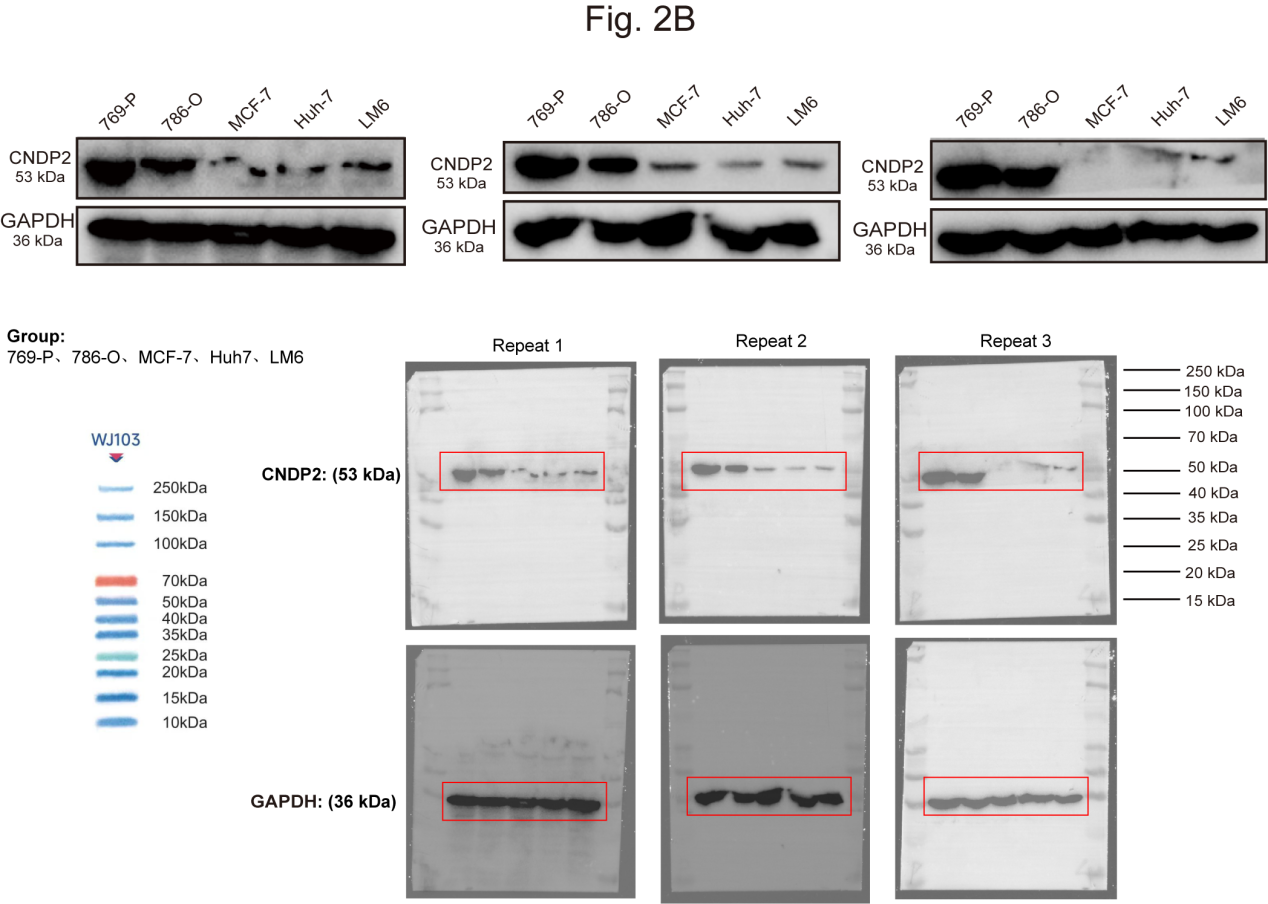


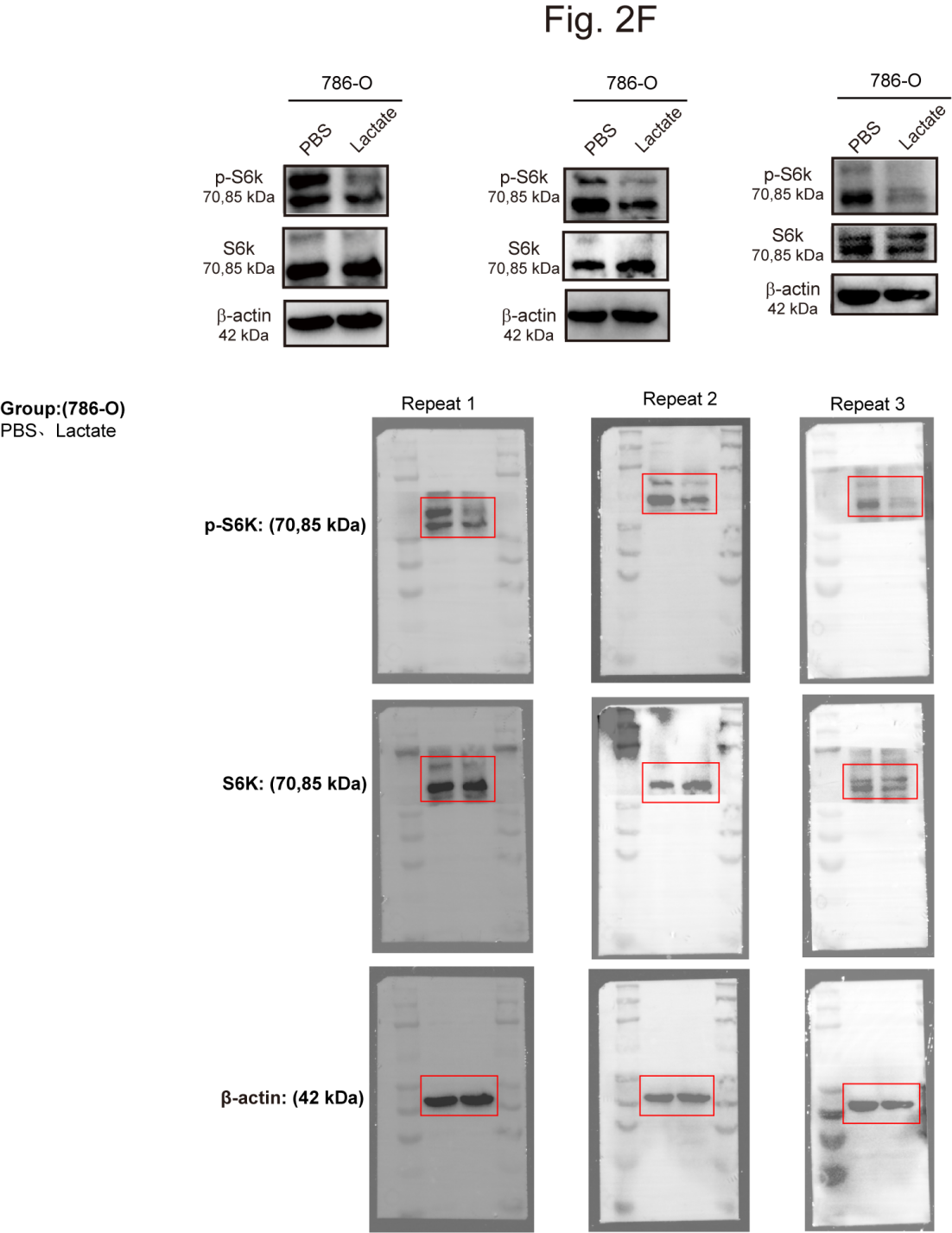


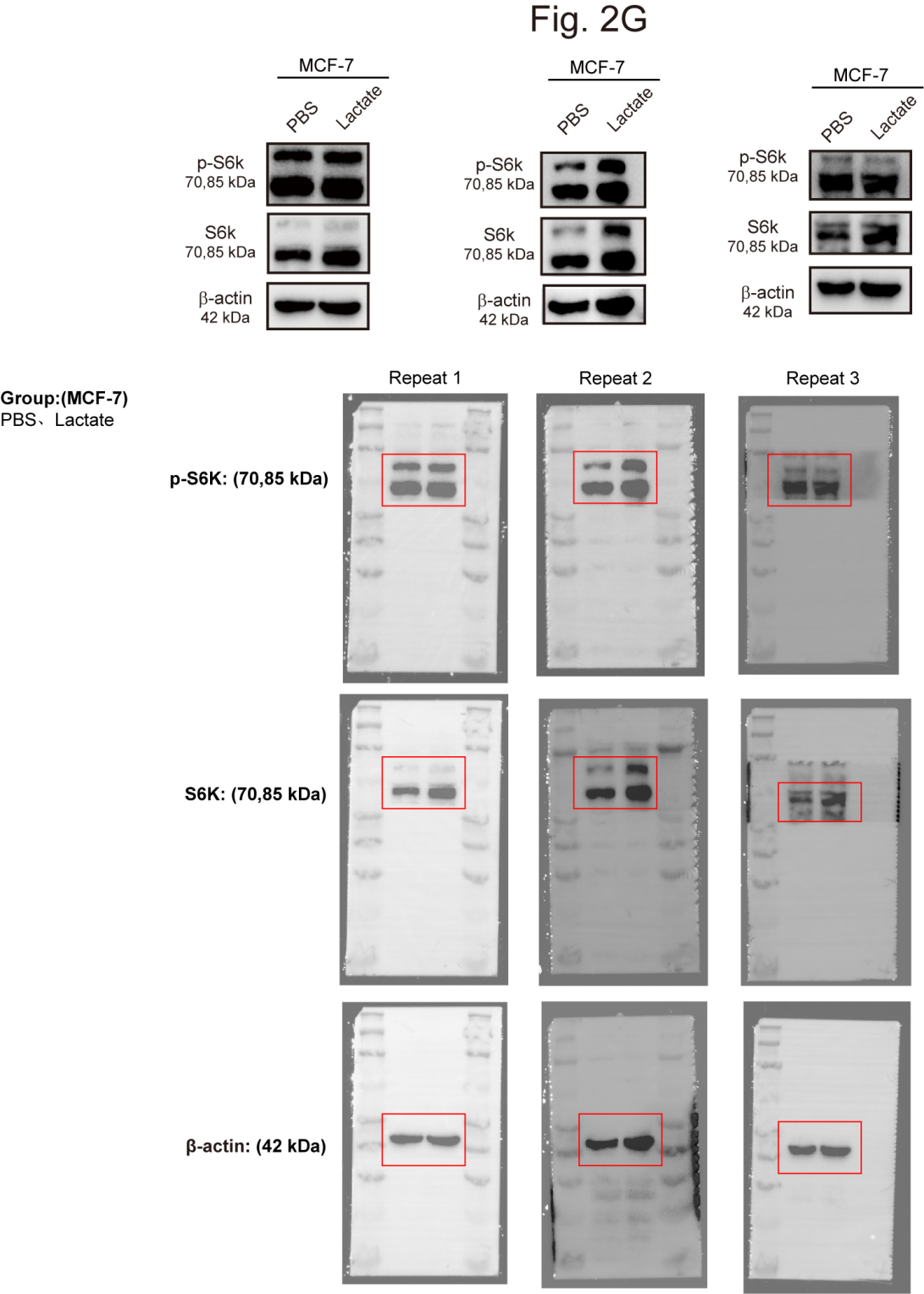


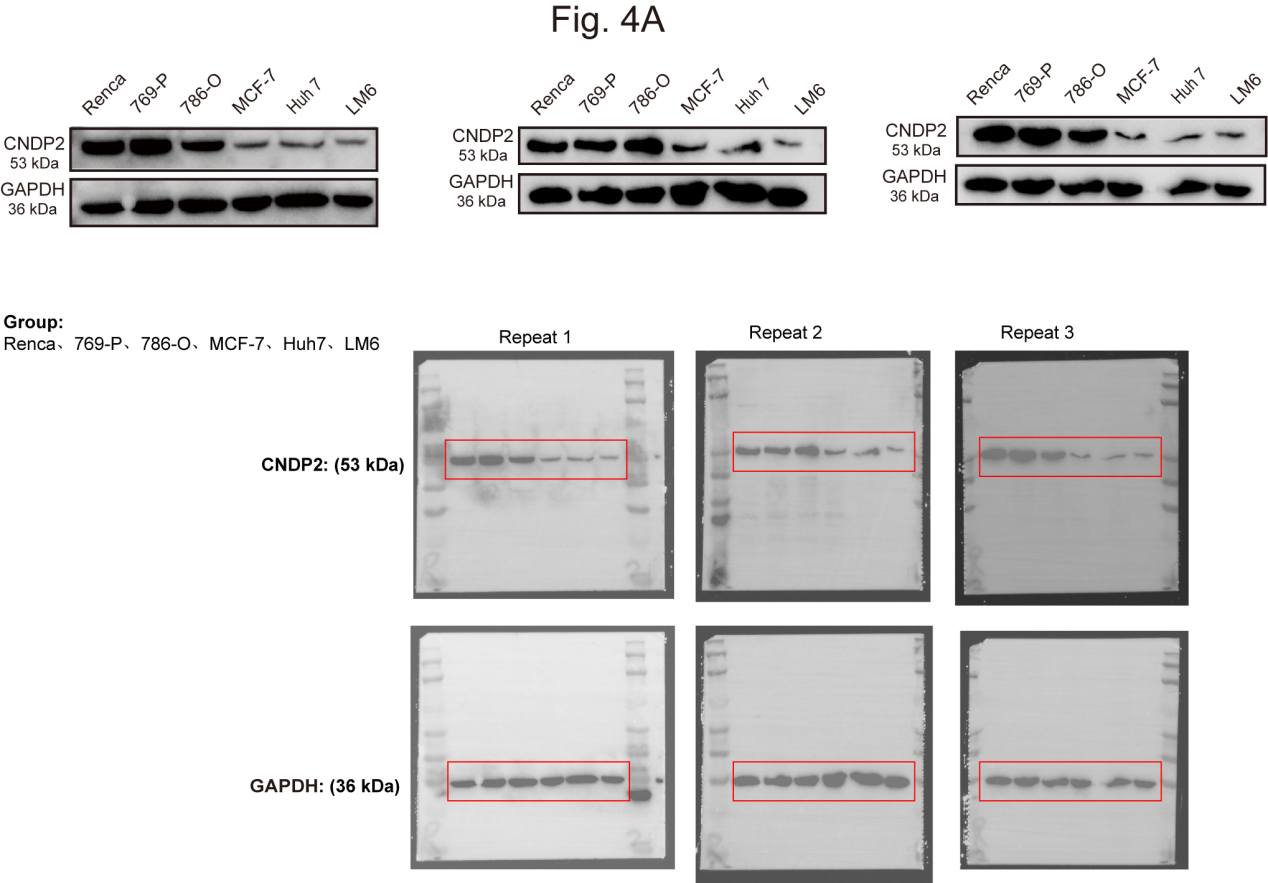


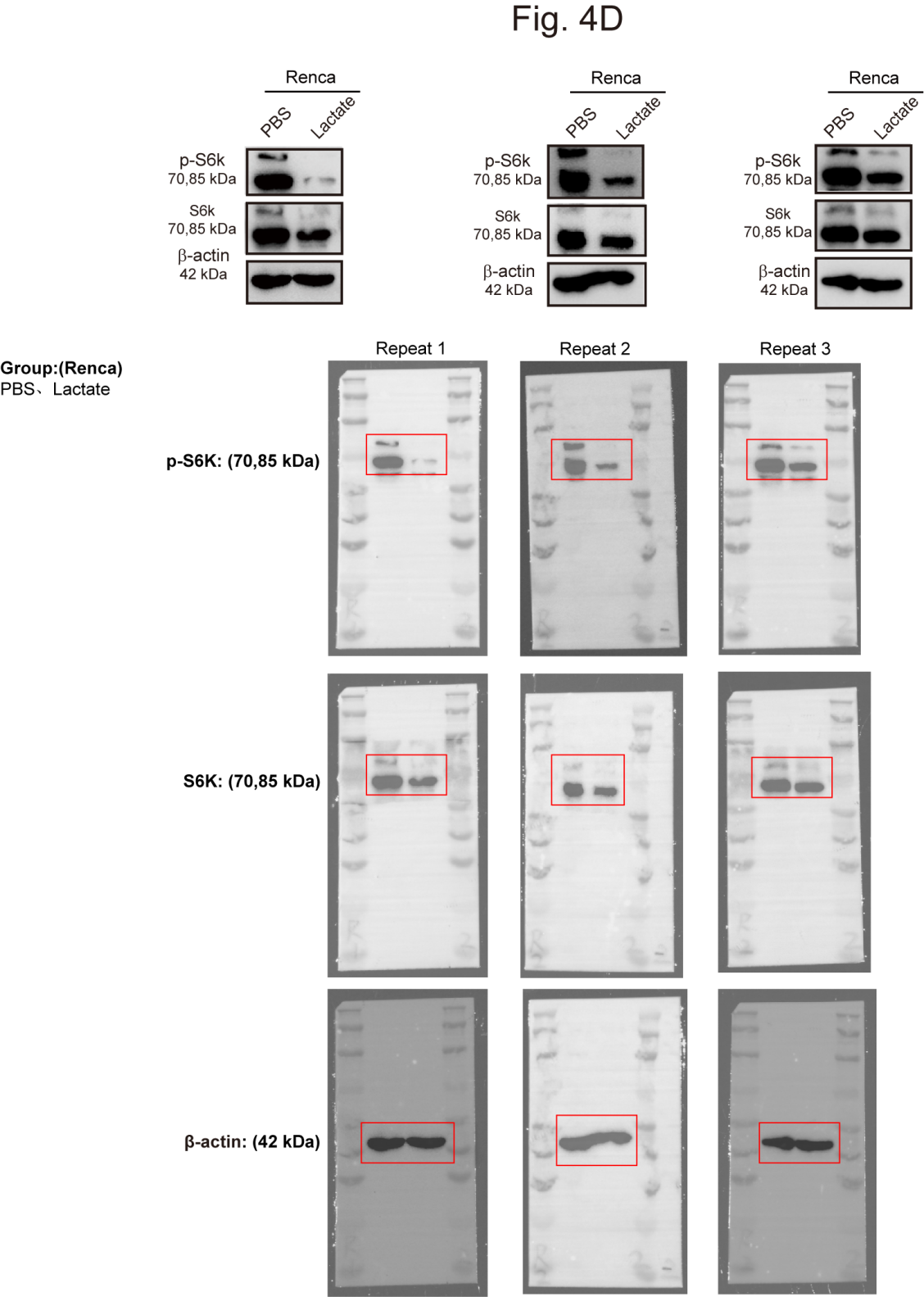


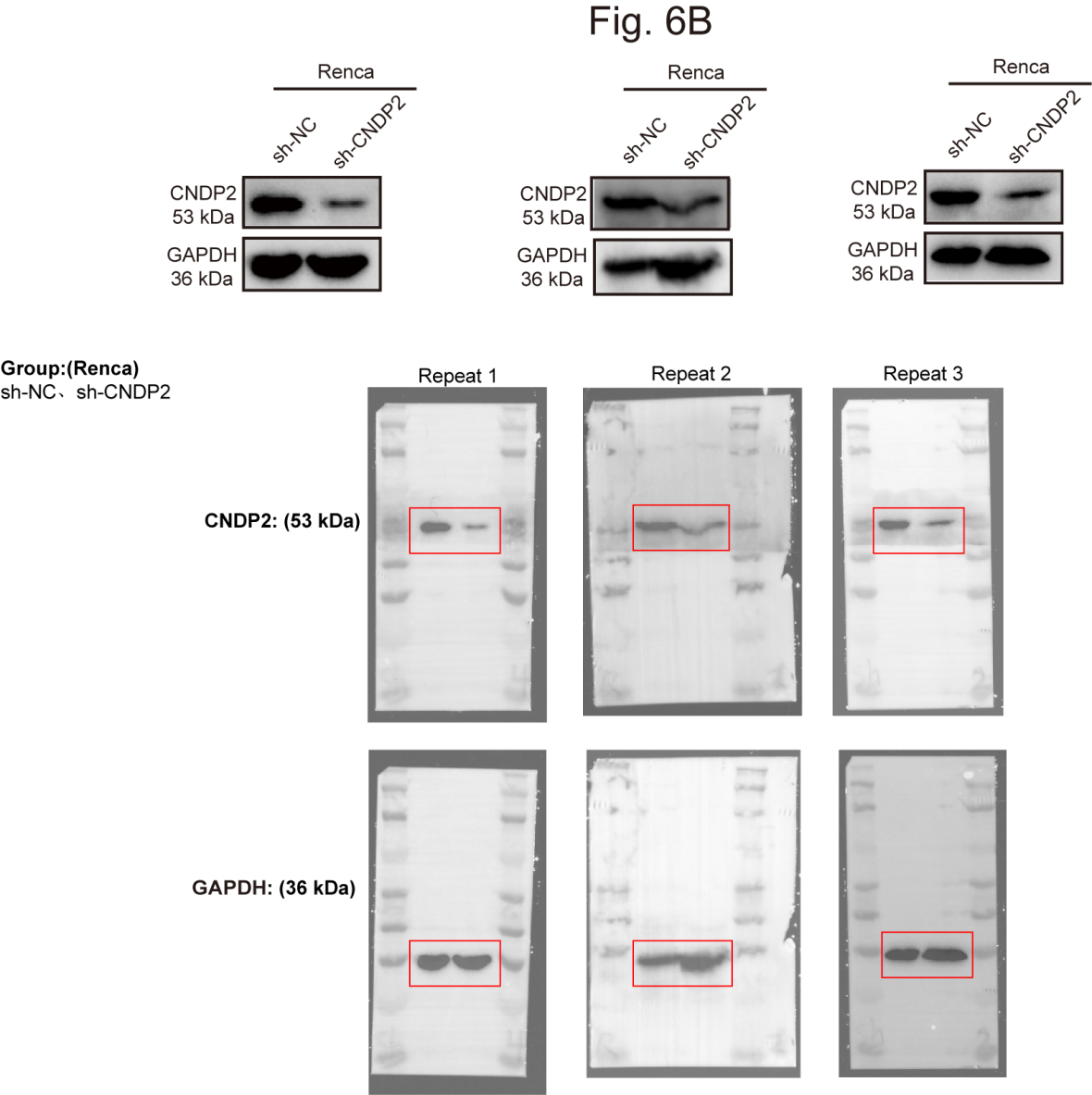


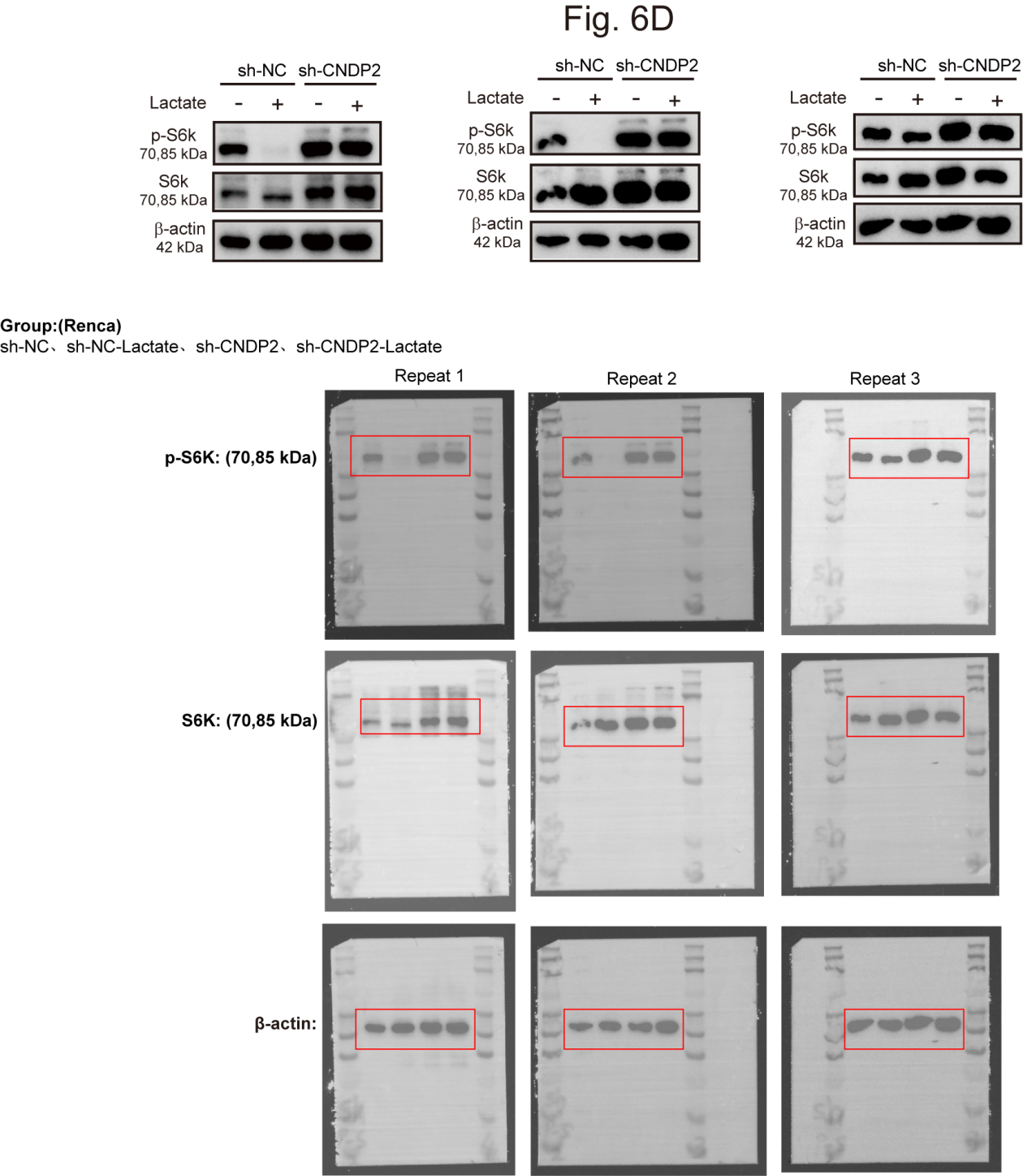


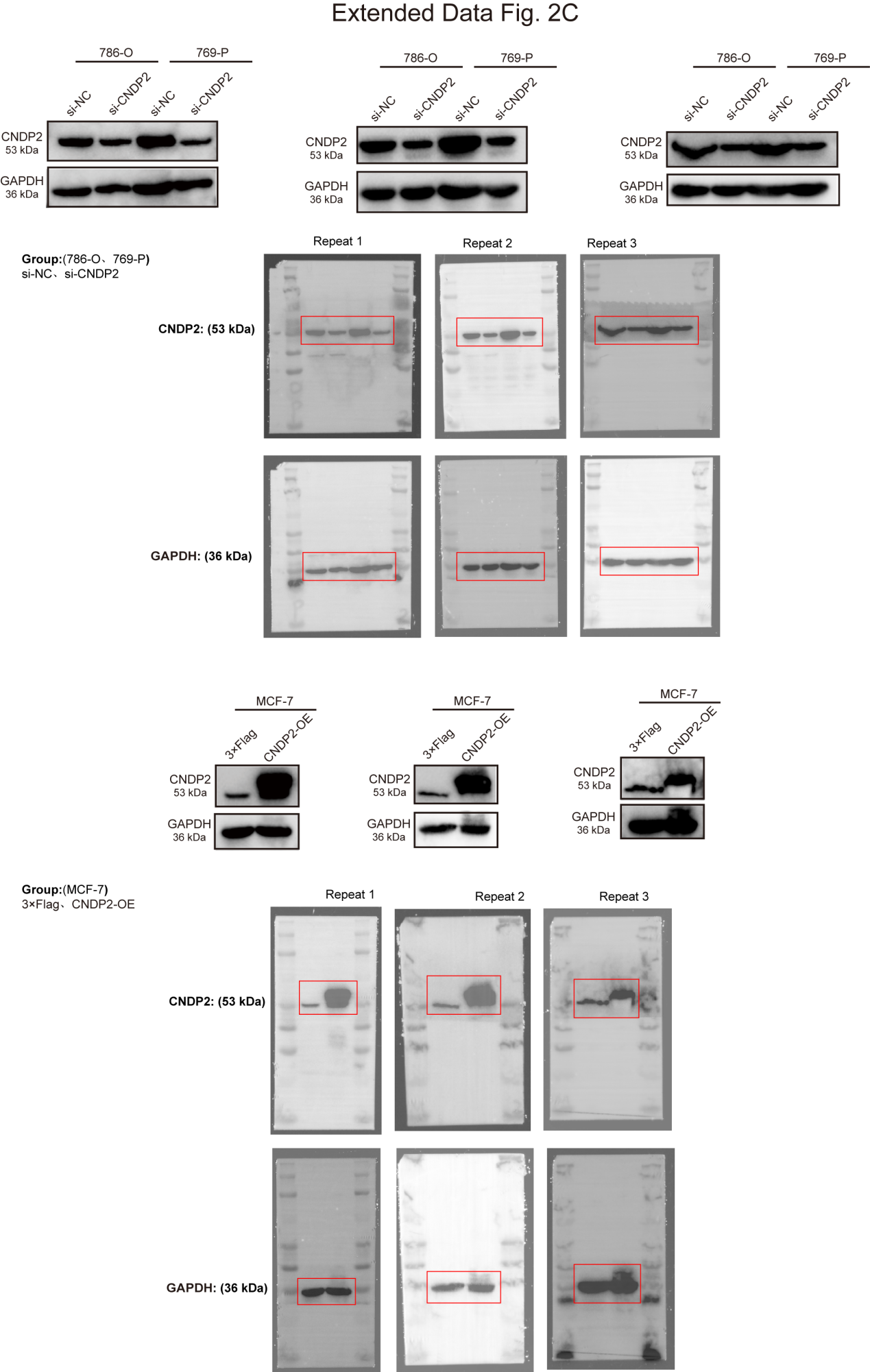

Supplement: Supplementary file 1 — Full and uncropped western blots [file 41420_2025_2609_MOESM1_ESM.docx]
