# Supplementary material for: Exercise-induced lactate suppresses ccRCC via CNDP2-mediated depletion of intracellular amino acids
Source: Cell Death Discov. 2025 Jul 31;11:356. doi: 10.1038/s41420-025-02609-3 (PMC12310999; doi:10.1038/s41420-025-02609-3)
Supplement: Supplementary file 2 — Supplementary Figure Legend [file 41420_2025_2609_MOESM2_ESM.docx]

**Supplementary Figure Legend**

**Supplementary Figure 1 ccRCC had the highest level of LDHA expression in 33 cancer tissues and was positively correlated with patient prognosis.**

**A,** Expression data of LDHA in 33 types of cancer and adjacent normal tissues were obtained from the GEPIA database. **B,** The correlation analysis between the expression levels of glycolysis and lactate transport-related proteins and the overall survival rate of ccRCC patients, as shown in the GEPIA database, is presented (n = 258).

**Supplementary Figure 2 Lactate can inhibit the proliferation of CNDP2+ ccRCC.**

**A,** The proliferation ability of 769-P and MCF-7 cells treated with lactate (15 mM) was determined by plate colony formation assay. The data were repeated three times independently. **B, C,** qRT-PCR and western blot were used to verify the silencing efficiency of CNDP2 at both the mRNA and protein levels in 786-O and 769-P cell lines, as well as the overexpression efficiency of CNDP2 at both the mRNA and protein levels in MCF-7 cells. The data were independently repeated three times. Data were analysed using two-tailed unpaired Student’s t test (ns, P > 0.05, *P < 0.05, **P < 0.01, ***P < 0.001 , ****P < 0.0001). **D,** The cell proliferation ability was assessed by detecting the content of Ki67 (red), and DAPI (blue) was used for nuclear staining. scale bar: 20 µm.
